# Fatigue Management in Multiple Sclerosis

**DOI:** 10.1007/s11940-016-0411-8

**Published:** 2016-04-18

**Authors:** Carmen Tur

**Affiliations:** National Hospital for Neurology and Neurosurgery, Queen Square, London, UK; Queen Square MS Centre, UCL Institute of Neurology, University College London, London, UK

**Keywords:** Multiple sclerosis, Fatigue, Disability, FSS, MFIS, VAS, Multidisciplinary approach, Exercise, Cognitive behavioural therapy, Energy conservation education programmes, Mindfulness intervention, FACETS, EXIMS, NICE guidelines

## Abstract

Multiple sclerosis (MS) is an inflammatory-demyelinating disease of the central nervous system that may entail severe levels of disability in the long term. However, independently of the level of disability, MS patients frequently experience severe fatigue that can be as disabling as objective neurological deficits. For that reason, it is mandatory to perform an early diagnosis of MS-related fatigue and start a suitable treatment as soon as possible. In clinical practice, MS-related fatigue should be assessed and managed by a multidisciplinary team involving neurologists, MS nurses, occupational therapists, and physiotherapists. When assessing a person with MS-related fatigue, the first step is to rule out potential triggers or causes of fatigue, which may be related to MS, such as urinary dysfunction, pain, or muscular spasms leading to a sleep disorder, or unrelated to it. Once these causes have been ruled out and appropriately tackled, a careful therapeutic intervention needs to be decided. Therapeutic interventions for MS-related fatigue can be pharmacological or non-pharmacological. Regarding the pharmacological treatments, although many drugs have been tested in clinical trials, only amantadine is currently recommended for this indication. Regarding the non-pharmacological approaches, they can be broadly divided into physical, psychological, and mixed physical/psychological interventions. Several studies, many of them randomised clinical trials, support the use of all these types of non-pharmacological interventions to treat MS-related fatigue. Recent publications suggest that the implementation of mixed approaches, which have a naturally comprehensive nature, may have excellent results in clinical practice, in relation not only to fatigue levels but also to more general aspects of MS.

## Introduction

Multiple sclerosis (MS) is an inflammatory-demyelinating disease of the central nervous system that is considered one of the most frequent causes of disability in the young adult [1]. In 85 % of the cases, MS starts as a relapsing-remitting disease. In this form of the condition, the first clinical attack, called *clinically isolated syndrome*, is followed by a number of relapses, after which there is generally a good recovery. After 15–20 years of symptom onset, there may appear a progressive clinical deterioration that is not related to the presence of clinical relapses, which is called secondary progressive MS (SPMS). In around 15 % of the cases, MS starts as a progressive condition called primary progressive MS (PPMS) [2].

Neurological disability in both progressive forms of MS mainly occurs at the expense of a progressive spastic paraparesis, which may eventually affect the upper limbs, and which can be accompanied by bladder and bowel dysfunction. Besides, a progressive brainstem/cerebellar syndrome may also be observed. Yet, apart from all these objective neurological deficits, disability in MS can also appear as a consequence of rather subjective symptoms such as fatigue [3•, 4•].

Fatigue in MS is extremely common. It may affect up to 80 % of the people with MS [5], and can be severe in up to 65–70 % of them [6, 7]. Importantly, it tends to persist over time once it appears [8]. Up to now, there is no consensus for the definition of fatigue, although Lapierre and Hum recently defined it as “subjective lack of physical and/or mental energy that is perceived by the individual or caregiver to interfere with usual or desired activities” [9]. It can have an important impact on the quality of life of people with MS, and, in some cases, can be perceived as disabling as loss of power in the limbs or walking issues. Of note, fatigue does not appear more frequently in those people with progressive forms of MS, especially PPMS, but instead it is more commonly reported amongst non-stable patients with relapse-onset MS [7], suggesting that its appearance is not necessarily related to objective neurological progression [8].

Fatigue in MS can be broadly divided into primary and secondary fatigue [3•]. Primary fatigue refers to that fatigue that appears without an apparent cause and it is specific of MS. Instead, secondary fatigue appears as a consequence of another condition, even if that is related to MS, and could theoretically appear in any other condition different from MS [10].

The pathological processes underlying MS-related fatigue are not yet well known. Some of the mechanisms proposed include structural abnormalities in deep grey matter regions [11, 12] and dysfunction of brain networks involving deep grey matter and cortex [11, 13, 14]. A recent review paper suggested that the fact that all these grey matter structures were associated with MS-related fatigue could be pointing at an imbalance in dopamine metabolism [15]. However, this hypothesis needs to be confirmed. Since a better understanding of these mechanisms will imply greater chances of finding effective treatments, more research in this field is guaranteed.

## Clinical aspects of MS-related fatigue

In clinical practice, the first step is to investigate whether there is an underlying cause or trigger for the fatigue, which needs to be treated before starting any specific treatment for it. Causes and triggers of fatigue may be related to MS, such as pain, night spasms and urine dysfunction, which can lead to sleep disturbances and increased tiredness, or being unrelated to MS, as it is described in Table 1.

**Table 1:** Possible causes and common triggers of fatigue in people with MS

Causes	Clinical red flags	Diagnostic procedures
Causes related to MS		
Depression	Low mood	Neuropsychological assessment
Sleep disorders	Excessive sleepiness, clinical features of conditions leading to sleep disorders, such as anxiety sleep apnoea, obesity	Assessment by sleep disorders specialist
Pain, muscular spasms	Pain or increased muscular tone during examination	Anamnesis, clinical examination
Bladder dysfunction such as nocturia, and urinary tract infections	High temperature	Temperature assessment, urine culture, assessment by the urologist or the bladder nurse specialist
Medication side effects	Recent start of a new drug or increase dose of previous medication	Anamnesis
Causes not related to MS		
Anaemia	Pale skin/conjunctivae	Determination of haemoglobin levels in blood
Thyroid dysfunction: hypo/hyperthyroidism	Recent changes in weight, hair loss, blood pressure	Determination of thyroid hormone levels in blood
Medication side effects	Recent start of a new drug or increase dose of previous medication	Anamnesis

Once possible underlying causes of fatigue have been ruled out, fatigue must be tackled through a multidisciplinary approach, involving neurologists, MS nurses, occupational therapists and physiotherapists. This multidisciplinary team will quantify the level of fatigue, which will be useful for future treatment monitoring. This team will also identify the best combination of therapeutic options for each individual, based on the severity of the fatigue and the presence of comorbidities.

Quantification of the level of fatigue and its impact on day-to-day life can be done through different scales (Table 2). The most commonly used are the Fatigue Severity Scale (FSS) [16] and the Modified Fatigue Impact Scale (MFIS) [17], in the setting of both clinical practice and clinical trials [18••]. Whereas FSS can be answered very quickly (only 9 questions), which may be very convenient in clinical practice, MFIS requires a bit more time (21 questions) but can give a more precise description of the impact of the fatigue on the subject’s day-to-day activities. For example, the questions contained in MFIS can be divided into three categories: physical, cognitive, and psychosocial. Therefore, for a given subject, not only a global fatigue score will be given but also separate scores for these three domains. These different scores may be useful to monitor therapeutic interventions, which may be more oriented to tackle certain domains of the fatigue than others [17]. Another scale of fatigue that has been frequently used in clinical trials is the visual analogue scale for fatigue, which is easy to administer and allows a very rapid assessment of fatigue [19]. However, its interpretation may be more difficult than that of more structured scales, and its reproducibility has proved to be only moderate, with an intraclass correlation coefficient below 0.7 [19].

**Table 2:** Commonly used fatigue scales

Scale	Reference	Description	Comments
Fatigue Severity Scale (FSS)	Krupp et al. *Arch Neurol* 1989	9 questions, with scores from 1 (strongly disagree) to 7 (strongly agree)	Easy to answer Available online
Modified Fatigue Impact Scale (MFIS)	Fisk et al. Canadian Journal of Neurological Sciences 1994	21 questions, with scores from 0 (never) to 4 (almost always), which can be divided into 3 categories: physical, cognitive, and psychosocial	Easy to answer Available online Provides information on 3 dimensions of fatigue: physical, cognitive, and psychosocial
Visual Analogue Scale (VAS) for fatigue	Kos et al. *BMC Neurol* 2006	10 cm straight line with equally distant numbers from 0 to 10 printed, where the individual must indicate to what extent fatigue is severe (0 = very severe, 10 = no fatigue)	Easy to answer It may be difficult to interpret (no specific domains of fatigue are explored)

Apart from the scales designed to specifically measure fatigue, there are some other, more general, scales that contain some questions to assess fatigue amongst other aspects of the underlying condition, such as the Expanded Disability Status Scale (EDSS) [20] or the Guy’s Neurological Disability Scale (GNDS) [21]. These scales can be useful to give an overall measure of disability, and, in particular, the EDSS has been—by far—the most commonly used disability scale in MS, in both clinical practice and clinical trials. However, they are not ideal to measure and monitor fatigue and other, more specific, scales are preferred. Finally, the SF-36 scale (i.e. the MOS 36-item short-form health survey) [22], a general scale to measure quality of life in people not necessarily diagnosed with MS, has also frequently used in clinical trials for MS fatigue, especially in relation to its items related to physical activity [23].

## Pharmacological treatments

Pharmacological treatments for fatigue in MS are summarised in Table 3. Amongst them, the most commonly used is amantadine. Its main mechanism of action is not yet fully understood, although its effects in fatigue seem to be related to its dopaminergic effects, supporting the dopamine imbalance theory for MS-related fatigue abovementioned [15]. To date, at least seven randomised clinical trials (RCTs) have compared amantadine with placebo [24–30] and one RCT has compared amantadine with aspirin [31]. In general, all trials that compared amantadine with placebo showed a significant effect of amantadine on fatigue. However, the results of these trials need to be taken with caution due to the low number of participants included in the trials, i.e. between 22 and 86 participants per trial, and the short duration of the interventions, which lasted between 3 and 6 weeks. The clinical trial that compared aspirin (500 mg daily orally) with amantadine in people with MS-related fatigue did not show differences between the two drugs. This trial, which had a crossover design and included 54 MS patients, showed a similar significant ability to decrease the levels of fatigue over time for amantadine and aspirin [31]. Importantly, since there was no placebo group, the results of this trial are difficult to interpret and require further research to better characterise the role of these two drugs in MS-related fatigue [31].

**Table 3:** Pharmacological treatment of MS fatigue

Drug	Common daily dose (maximum daily dose), and route	Mechanism of action	Quality evidence from clinical trials^a^	Main side effects
Amantadine	100 mg twice a day (400–600 mg/day), oral	Dopaminergic effect	Low to moderate	Insomnia, dizziness, headache, hallucinations, peripheral oedema, livedo reticularis, orthostatic hypotension, dry mouth, anorexia, nausea, constipation
Modafinil	100 mg twice a day (400 mg/day), oral	Probable dopaminergic effect (atypical dopamine reuptake inhibitor)	Very low	Insomnia, nervousness, dizziness, headache, nausea, asthenia
4-aminopyridine	10–20 mg/day (60 mg/day), oral	Potassium channel blocker	Low for fatigue High for walking speed	Epileptic seizures, insomnia, anxiety, dizziness, paraesthesia, tremors, headache and asthenia
Antidepressants (paroxetine)	10–40 mg/day (60 mg/day), oral	Selective serotonin reuptake inhibitor (SSRI)	Very low	Increases in cholesterol levels, decreased appetite, somnolence, insomnia, agitation, nightmares, suicidal ideation, concentration impaired, dizziness, tremor, headache, blurred vision, nausea, constipation, diarrhoea, dry mouth, sweating, sexual dysfunction, asthenia, body weight gain Abrupt withdrawal must be avoided
L-Carnitine	1 g twice a day (not clear), oral	Amino acid derivative involved in the metabolism of lipids and mitochondrial energy production	Very low	Side effects are very rare and may include nausea, diarrhoea, abdominal cramp, and increase in International Normalised Ratio (INR)

^a^Based on the number of clinical trials, the number of participants per trial, and the results obtained in the different trials

The daily dose of amantadine used in all published trials was 200 mg, which is the now accepted dose of amantadine for MS fatigue [18••]. Of note, amantadine is the only oral treatment that is currently recommended by the National Institute for Health and Care Excellence (NICE) for the treatment of MS-related fatigue [18••].

Modafinil, a drug currently approved for the treatment of attention-deficit hyperactivity disorder (ADHD) and narcolepsy, has also been frequently evaluated in clinical trials for patients with MS-related fatigue. In 2002, Rammohan et al. published a 9-week single-blind clinical trial where 72 patients received placebo for the first 2 weeks, modafinil 200 mg daily for the following 2 weeks, modafinil 400 mg daily for the following 2 weeks, and again placebo for the final 3 weeks [32]. This trial showed a significant improvement in fatigue scores when patients were receiving modafinil 200 mg per day, although no significant treatment effect was seen during the period when patients were receiving modafinil 400 mg per day. A few years alter, a randomised placebo-controlled double-blind clinical trial with a parallel arm design and carried out with 110 patients showed no significant effect of modafinil (up to 400 mg daily), given during a period of 5 weeks, on MS-related fatigue [33]. In 2009, another randomised placebo-controlled trial evaluating the effect of modafinil and carried out in 21 MS patients was published. This time, the drug, which was given during 8 weeks, showed a significant effect not only on fatigue scores but also on a motor task of the upper limbs (the 9-hole peg test [34]), and a cognitive test. Interestingly, it also showed an increase in the amplitudes of motor evoked potentials, suggesting modafinil might have an effect at the level of motor cortex function [35]. However, in 2011, the results of a much larger trial, which included 121 MS patients and also evaluated modafinil 200 mg daily over 8 weeks, showed, in general, negative results [36]. Finally, in 2015, a very small trial again showed no effect of modafinil on fatigue scores, although some effects on cognitive outcomes were observed [37]. Thus, the overall evidence supporting the effect of modafinil on MS-related fatigue is weak, and in the last edition of the NICE guidelines modafinil was not included as a recommended treatment for MS-related fatigue [18••].

Pemoline, a drug with some similarities with modafinil for its dopaminergic effects and its initial approval for the treatment of ADHD and narcolepsy, has also been tested in clinical trials for MS-related fatigue, with overall negative results [26, 27, 38]. Besides, it has been withdrawn from the market and is no longer available for ADHD or narcolepsy either due to its side-effect profile, since it has been associated to fatal liver failure.

The effect of 4-aminopyridine and its related compound, 3,4-diaminopridinine on different aspects of MS has been frequently assessed over the last 25 years. The 4-aminopyridine (also known as fampridine) is a potassium channel blocker and therefore increases the duration of the action potential, which translates into an increase in muscle strength. Apart from this peripheral effect, some authors have also proposed a central effect, after observing an increase in the bold signal of a motor-task functional magnetic resonance imaging performed after the administration of the related compound 3,4-diaminopridinine, as compared to that observed after the administration of placebo [39].

The evaluation of the efficacy of 4-aminopyridine on different symptomatic aspects of MS, including MS fatigue, started around 25 years ago [40–43]. In 1992, a clinical trial comparing 4-aminopyridine and placebo was published. In this trial, which included 70 participants, those who took active treatment showed an improvement on the EDSS and a subjective improvement of fatigue levels, with positive consequences in the ability to carry out day-to-day activities [42]. During the following years, the results of this trial were somehow confirmed, but only in non-randomised studies [40, 41]. In 2001, the results of a new randomised placebo-controlled trial double blind clinical trial were published. This was a small crossover trial, with 54 individuals included, where the effects of 4-aminopyridine were evaluated. This trial did not show significant differences between active and placebo arms in terms of fatigue scores, EDSS and cognitive scores. Interestingly, though, it did show an improvement in the fatigue levels of those subjects with the highest blood levels of 4-aminopyridine, implying a dose-response relationship, a key element to support a therapeutic effect on fatigue levels, as had been suggested in previous studies [43]. However, in the clinical trials that have been published more recently, it seems that this drug, although effective in improving walking speed, does not have a clear effect on fatigue [44–46].

4-aminopyridine is considered to be quite safe and well tolerated. However, some side effects, mainly neurological, may appear. These include insomnia, anxiety, dizziness, paraesthesia, tremors, headache, asthenia and epileptic seizures [44–48]. Apart from these, other side effects might also appear. For that reason, before starting this or any other drug, it is always convenient to check the document issued by the European Medicines Agency (EMEA) describing the summary of the product characteristics [49].

Antidepressants have also been tried for the treatment of fatigue in MS. In particular, a RCT compared paroxetine with placebo [23], showing some beneficial effects of paroxetine as compared with placebo in terms of the mental subscale of SF-36, and the cognitive and psychosocial subscales of MFIS. However, the results of this trial need to be interpreted with caution given that only 42 patients were enrolled in the study and also because paroxetine did not seem to have an effect on the physical scales of the SF-36 and the MFIS [18••]. On the other hand, paroxetine is quite a well-tolerated drug [50], whose main side effects are shown in Table 3.

L-carnitine has also been evaluated to treat MS-related fatigue. It is an amino acid derivative involved in the metabolism of lipids and is a key element in mitochondrial energy production [51]. For this reason, it was believed it could improve fatigue levels in patients with chronic conditions [52]. At least three studies have evaluated the efficacy of L-carnitine in MS-related fatigue [28, 53, 54], although only two of them were randomised trials [28, 53]. In the first one of these two trials [53], which had a crossover design, acetyl L-carnitine 2 g/day was compared to amantadine 200 mg/day in a group of 36 patients (30 finished the trial). Treatments were given for 3 months to all patients, with a washout period between them of also 3 months. Although this trial showed some beneficial effects of L-carnitine as compared to amantadine with regard to FSS scores, the fact that no placebo group was used together with the low number of participants enrolled imply that the results must be taken with caution [51]. The second one of these trials [28], which had a parallel arm design and included 60 patients, compared L-carnitine 2 g/day, amantadine 200 mg/day, and modafinil 200 mg/day with placebo in terms of their ability to improve MS fatigue. At the end of the trial, only amantadine showed a significant beneficial effect versus placebo. Although the group on L-carnitine had better scores than the placebo group, the differences were not significant, maybe indicating lack of power. Instead, the group on modafinil showed very similar fatigue scores to the placebo group, suggesting no beneficial effect of modafinil [28]. Again, the evidence supporting L-carnitine to treat MS-related fatigue is weak, and this treatment is not currently recommended by NICE guidelines for this purpose [18••].

Other pharmacological approaches have also been mentioned in the literature, such as vitamin B12 intramuscular injections. However, in the NICE guidelines, there is a clear recommendation of not offering this or other drugs for which scientific evidence is lacking to treat MS-related fatigue [18••].

## Non-pharmacological treatments

Once triggers of fatigue are identified and tackled [55], it is crucial to set relevant and achievable goals before starting any therapeutic intervention. Generally, the multidisciplinary team, especially the occupational therapists and physiotherapists, will help the patient to set these goals. In many cases, these goals will be related to instrumental activities of day-to-day life. Besides, they will need to be rather short-term goals in order to make their achievement more feasible [56]. Finally, a convenient non-pharmacological intervention will be agreed for a given individual. These interventions can be broadly divided into physical, psychological/cognitive, and mixed approaches [18••].

Amongst the physical approaches, one of the most frequently assessed has been aerobic exercise. In 1992, the results of a very well designed trial were published. It included 54 MS patients, which were randomly divided into two groups: exercise vs. no exercise. The intervention lasted for 15 weeks, and the group assigned to exercise improved on almost all clinical scores and biological markers except for the FSS scores, which remained unchanged for both groups throughout the trial [57]. However, a more recent trial did show beneficial effects for aerobic exercise as compared to placebo on MS fatigue. In particular, this trial compared physiotherapist-led exercise, fitness instructor-led exercise, yoga, and placebo (no particular intervention) and showed that all three interventions were effective (as compared with placebo) in improving fatigue. Additionally, the two exercise interventions showed also an effect on scores related to objective physical disability [58]. Apart from these two trials, a number of studies have been carried out and a recent Cochrane review has concluded that there is a significant effect of exercise on fatigue outcomes in people with MS [59••]. Besides, a recent study has found an association between the improvement in fatigue levels after regular exercise in people with MS and changes in the expression of genes related to modulation of systemic response to interferon [60]. Although the results of this study need to be replicated in larger cohorts, it provides a possible biological explanation for the beneficial effect of exercise in MS [60].

Other physical approaches that have been evaluated include resistance training, electromagnetic field therapy, and cooling therapy. Whereas resistance training has shown a clear significant effect against placebo on MS-related fatigue [18••], the evidence behind the other two approaches is too low to emit recommendations in this regard [6, 61].

The psychological/cognitive approaches include cognitive behavioural therapy, energy conservation education programmes and fatigue management, and mindfulness intervention. Cognitive behavioural therapy aims to address behaviours of people with MS in order to improve their levels of fatigue. Its efficacy has been proved in several studies and the NICE guidelines recommend its use, either alone or in the context of more comprehensive programmes with other non-pharmacological approaches [18••]. Regarding energy conservation education programmes and fatigue management, these are approaches that try to help he patient to save energy through the implementation of different strategies such as work simplification or the use of labour-saving and ergonomic equipment [3•]. Lastly, there are the mindfulness-based interventions, which are based on “an intentional and non-judgemental awareness of the present moment”, as has been described in the literature [62, 63]. They derive from Buddhist practice and have been used for a number of psychological and somatic conditions, including MS-related fatigue. For that reason, their use is currently recommended by the NICE guidelines, amongst other psychological approaches [18••].

Finally, there are the mixed approaches, which combine both physical and psychological interventions [3•]. The mixed programmes that are best known are the FACETS (Fatigue: Applying Cognitive Behavioural and Energy effectiveness Techniques to lifeStyle) [64••, 65••] and the EXIMS (pragmatic EXercise Intervention in people with MS) [66••]. The FACETS was a very well designed randomised placebo-controlled trial where 164 patients with MS were included and randomly assigned to FACETS plus usual care (current local practice) or to usual care only. This study showed a beneficial effect of the FACETS programme, which consisted of six weekly sessions of around 90 min, on fatigue levels. Interestingly, this beneficial effect was observed after 1 and 4 months after finishing the intervention [64••] and was still maintained at 1 year of follow-up [65••]. Similarly, the EXIMS study was carried out in 120 patients with MS, who were randomly assigned to a 3-month exercise intervention plus usual care or to usual care only. Its primary objective was to evaluate the effect of a combined physical and psychological programme on the self-reported exercise behaviour. Remarkably, the authors found that the programme not only significantly improved the exercise behaviour but also was associated with a decrease in fatigue levels [66••]. In relation to these results, it is worth mentioning that the current NICE recommendations for patients with MS and in particular with MS-related fatigue include not only physical or cognitive interventions but also the promotion of long-term exercise [18••].

## General considerations and conclusions

Fatigue is one of the most disabling symptoms in MS and needs an accurate diagnosis and a rapid intervention. The assessment and treatment of MS-related fatigue may be complex, and it is mandatory to rule out potential causes or triggers of fatigue. It is important to bear in mind that these causes may or may not be directly related to MS so careful evaluations are mandatory.

Once primary causes of fatigue are ruled out, a multidisciplinary assessment is necessary to ensure the patient receives the best therapeutic approach. Although many drugs have been tested in multiple clinical trials, only those trials with amantadine have provided enough evidence to allow the recommendation of this treatment for MS-related fatigue. Yet its effect is moderate and the quality of that evidence may be considered as low to moderate, according to the NICE guidelines. Undoubtedly, the scarcity of oral treatments able to improve the levels of MS-related fatigue in a satisfactory manner reflects our poor understanding of the processes underlying fatigue in MS. Thus, more research into this disabling symptom of MS is warranted.

Amongst all non-pharmacological approaches, the mixed physical and psychological approaches deserve especial attention. They are naturally comprehensive, and their corresponding clinical trials have shown promising results, which may translate into excellent results when applied in clinical practice. These mixed approaches emphasise once again the need for multidisciplinary teams and assessments. This should be taken into account when new units specialised in MS and other neurodegenerative disorders are being designed. Besides, the newly appeared mixed approaches promote other important aspects of the MS care, such as the performance of regular exercise, which can have benefits potentially beyond the improvement of MS-related fatigue.
